# Hospital and Health News

**Published:** 1923-10

**Authors:** 


### HOSPITAL AND HEALTH NEWS.

### Bequest to Swansea Hospital.

A working-man, the late Mr. David Evans, has bequeathed £1,796 to the Swansea Hospital to name two beds there.

### Taxis for Child Patients.

Mr. G. W. Potts, a Smithfield merchant, has given £500 to provide taxis to carry child patients home after minor operations at St. Bartholomew's Hospital, the *Journal* of that hospital says.

### A Beauty Competition.

A prize for the handsomest man was one of the attractions offered at a fête which took place recently on behalf of the Leicester Royal Infirmary at the village of Peckleton.

### Opening of a Sanatorium.

The new King Edward VII. Memorial Sanatorium at Hertford Hill, near Warwick, will be opened informally on October 6. At the official opening in 1924 a statue of King Edward VII. will be unveiled.

### Glove Inside a Man.

Surgeons who operated recently on a man for cancer at Salem, Oregon, discovered that a suspected malignant internal growth was a surgeon's rubber glove left inside him at an operation which took place at San Francisco ten years previously.

### New Knights of Grace.

Mr. Henry Seymour Berry, of Bwlch, Breconshire, and Surgeon Lieut.-Col. Evan Evans, J.D., M.B., F.R.C.S., D.P.H., of Llanelly (from an Honorary Associate), have been appointed Knights of Grace of the Order of St. John of Jerusalem in England.

### Lectures on Personal and Public Health.

A course of lectures on "Problems of Personal and Public Health" has been arranged by the Royal Institute of Public Health, and will be delivered in the Lecture Hall of the Institute, 37, Russell Square, W.C. 1, on Wednesday afternoons at 4 p.m., from October 17 to December 12.

### St. Mary's Hospital, Paddington.

The annual dinner of the past and present students of St. Mary's Hospital Medical School will take place at the Connaught Rooms, Great Queen Street, W.C., on Monday, October 1, 1923, at 7 for 7.30. Mr. V. Warren Low, C.B., F.R.C.S. will be in the chair. The Hon. Secretary is Dr. Hope Gosse.

### Wireless Appeal for Blood Transfusion.

The latest use to which hospital appeals by wireless have been put was one for blood transfusion on behalf of a patient at King's College Hospital. Thirty-five candidates responded in person, not to mention replies by telephone; and the appeal was broadcasted because none of the local volunteers on the hospital's register happened to be available.

### Refusing Sweepstake Money.

The Nursing Association is stated to have refused a percentage of the proceeds of a sweepstake because the association's committee thought that sweepstakes were morally wrong. But, according to a correspondent of *The Guardian*, a Church of England church in Staffordshire is raising money by means of a raffle. The correspondent, however, does not infer from this that the ecclesiastical view may be the right one.

### A Memorial Tablet.

A tablet has been placed in Richmond Royal Hospital to the memory of the late Mr. J. Culver, J.P., who collected for it in 1897, and for many years was chairman of the United Societies' Fete Committee, "in grateful recognition of his untiring zeal and unselfish devotion to the sick." At the unveiling, Colonel Leslie Powell related how, though a master-man and an employer, Mr. Culver had been recommended and appointed "a working-man Justice of the Peace" not very long before his death.

### New Secretary of Warneford Hospital.

The successor to the office of house governer at Warneford Hospital, Leamington, which, as stated in our September issue, Mr. C. R. W. Often resigned on September 8, is Mr. W.

Russell Rudall, of Stevenage, Hertfordshire. Mr. Rudall, who was chosen out of 350 candidates, is twenty-eight years of age, and before his previous appointment on the administrative staff of St. Bartholomew's Hospital, had previously belonged to the secretariat of St. Mary's and of King's College hospitals.

### A National Milk Conference.

The National Clean Milk Society has convened a National Milk Conference (subject: Pasteurization), to be held on Wednesday, November 21, in the Council Chamber at the Guildhall. Papers will be read on: Methods and Processes of Pasteurization; Physical Changes (Cream Line, etc.); Chemical Changes (Salts, etc.); Bacteriological Changes; Biochemical Changes; and the Financial and Commercial Aspect.

### The Edith Cavell Homes of Rest.

We are asked to state that there are only four houses in existence known as the Edith Cavell Homes of Rest for Nurses. They are The Hollies, Gypsy Road, West Norwood; The Crossways, Windermere; Raven House, Adderley, Market Drayton, and Coombe Head, Haslemere. All information regarding these Homes can be obtained from Miss Hall, 32, North Audley Street, W. 1.

### Another X-Ray Martyr.

Mr. R. Blackall, an X-ray worker at London Hospital, has had to undergo amputation of both hands. He is the third member of the hospital's X-ray staff to lose his hands in this manner. It is stated that at the present time there is ample protection for X-ray workers, but, unfortunately, in the three cases mentioned, the harm was inflicted before the danger came to be fully realised. Mr. Blackall had been engaged in X-ray work since 1900.

### Health Lectures in Prisons.

The People's League of Health, which, it will be remembered, inaugurated with the sanction of the Home Office in April, 1922, this important educational work in His Majesty's Prisons and Borstal Institutions, has arranged for lectures to be delivered at Oxford, Wormwood Scrubs and Holloway Prisons during this autumn. The lecturers will include Sir Bruce Bruce Porter, Sir James Cantlie, Sir John Collie, Professor C. S. Myers (President of National Institute of Industrial Psychology) and Professor A. Louise McIlroy.

### The Film Out-filmed at Guy's.

The September number of *Guy's Hospital Gazette* contains, in pica type, the following notice: ". . . . The next issue will contain the first instalment of a thrilling drama of real life entitled, "Pursued by Bandits," being the true story of the attempted victimisation of a Guy's Doctor by two of the most audacious and blood-thirsty rogues in the South of London . . . . from the moment when the victim was first entrapped to the daring and heroic rescue by picked men of the Hospital special constabulary, led by a clinical assistant."

### Lecture on Infant Care.

A course of elementary lectures on Infant Care, especially intended for crèche nurses and probationers, will be held in the Lecture Hall, Carnegie House, 117 Piccadilly, W. 1, on Thursdays, from 7.30 to 8.30 p.m., from October 4 to December 6. The lecturer will be Dr. Flora Shepherd, Medical Officer for Maternity and Child Welfare, Hornsey, and the course is in preparation for the Elementary Crèche Nurse's Certificate instituted by the National Association for the Prevention of Infant Mortality and the National Society of Day Nurseries. All particulars can be obtained from the secretaries of either of these societies at 117 Piccadilly, W. 1.

### Poplar Hospital Justified.

In reply to the allegation of neglect made by the jury at a recent inquest on a man admitted, dressed and then sent to the Mile End Hospital on an ambulance from the Poplar Hospital for Accidents, Mr. D. H. Lindsay, secretary and house governor has issued a statement. In regard to the seven beds empty at the time of the patient's arrival, Mr. Lindsay said that if the man's condition had indicated risk to his life by removal he would have been retained, that the empty beds were reserved for grave emergencies and used for them later on that same day. He added that this was the first case during thirty years in which death has followed refusal to admit at the Poplar Hospital for Accidents.

### Local Authorities and Cancer.

The Departmental Committee appointed by the Minister of Health have prepared a brief and non-technical memorandum on cancer for the guidance of Local Authorities. The memorandum gives some account of the characteristic features and natural cause of cancer, its symptoms, diagnosis and treatment, and emphasizes the need for anyone suspecting himself of cancer to instantly consult a doctor. With reference to propaganda the committee wisely points out that "much caution is obviously needed in announcements to the public on cancer in order to avoid over-statement, the making of promises which are not warranted by evidence, or the production of needless and mischievous apprehension."

### World's Dairy Congress.

The Government were invited by the President of the United States to take part in a World's Dairy Congress, to be held at Washington in October, under the auspices of an unofficial association with the patronage of the United States Government. The invitation has been taken up by the Ministry of Health and by the Ministry of Agriculture and Fisheries, and a committee, including representatives of the Departments, of Local Government Associations, and of organisations interested in the dairy industry, has been formed for the purpose of arranging for the representation of this country at the Congress and the submission of suitable papers. It has been decided that an officer of the Department shall attend the Congress.

### Thomas Guy's Tomb.

The tomb of Thomas Guy, in the crypt of the chapel of the hospital of which he was the founder, is being reopened for public inspection. Originally accessible through the matron's kitchen, the tomb is now approached by an entrance of its own. Next year will be the bi-centenary of Guy's death and of the foundation of the hospital. Apprenticed to book selling, in 1668, at the age of twenty-four, he eventually obtained the privilege of printing Bibles. By fortunate investment, including, it is interesting to recall, South Sea stock (through which Guy lost the fortune that he wrote *The Beggar's Opera* to repair), Guy gained great wealth. He built Guy's Hospital at a cost of £18,800 and endowed it with nearly a quarter of a million.

### Lectures at the Royal Sanitary Institute.

The Royal Sanitary Institute issues its prospectus of courses of training for sanitary officers, health visitors, school nurses, maternity and child welfare workers, beginning on September 25, at 6 p.m., and on October 1, at the same hour. These training courses are of particular interest just now, when so many educated women are being appointed on the staff of Public Health Authorities, and the demand for trained women appears to be increasing. The training not only includes lectures, but practical demonstrations in the Museum of Sanitary Appliances, at infant consultations and child welfare centres, visits to public works and other places of sanitary interest, and the use of a reference library, lending library and reading room. The lectures are followed by the standard examinations of the institute, which are recognised in all parts of the British Empire. All further information can be obtained from the director and secretary, Mr. E. White Wallis, at the Institute, 90 Buckingham Palace Road, S.W. 1.

### Scottish Nurses and Health Visitors.

The badge for registered nurses issued by the General Nursing Council for Scotland is now on sale and can be obtained from the manufacturers, Messrs. Brook and Son, 87, George Street, Edinburgh, at the price of 4s. 9d. (including postage), on production to Messrs. Brook and Son of a voucher, which must be obtained from the Registrar. To save correspondence, the Council are arranging to issue in future one of these vouchers to each nurse as she pays her retention fee for this year. Nurses who have not still to pay a retention this year can obtain a voucher by communicating with the Registrar at 13, Melville Street, Edinburgh, to whom the price of the badge is on no account to be remitted. The Scottish Board of Health announce the opening of their Register of Health Visitors. Persons discharging on behalf of a local authority all or any of the duties of a health visitor under a scheme of maternity service and child welfare or of tuberculosis or otherwise, and also persons discharging all or any of the duties of a school nurse under a scheme of school health administration may apply for certification and registration. Forms of application will be supplied by the Board, 121A Princes Street, Edinburgh.

